# 

**Published:** 1886-01

**Authors:** 


**Original Communications:** PAGE.

Expulsion of a Large Sub-mucous Fibrous Tumor. J. H. Etheridge. 1
Pelvic Abscess. H. T. Byford ..... 7

**Editorial:**

The American Public Health Association ..... 14
The Ninth International Medical Congress ..... 15

**Society Reports.**

*Chicago Medical Society.* Expulsion of a Large Sub-mucous Fibrous Tumor. J. H. Etheridge. 19
Vesicle Calculus. J. Franks ..... 21
December 7th, 1885. Pneumonic Abscess. E F. Wells ..... 21
*Chicago Gynecological Society. November 27th, 1885.* Toxic Properties of Sassafras. J. Bartlett ..... 24
Remarks on a Teratom. C. W. Earle. 26
Artificial Abortion ..... 27
*Chicago Society of Ophthalmology and Otology.* Ossification of the Choroid. L. Ware ..... 29
Choroiditis following Typhoid Fever. F. C. Hotz ..... 33
*College of Physicians of Philadelphia.* Cocaine in Hay-Fever. J. M. Da Costa ..... 35
Treatment of Sun-stroke. O. Horwitz ..... 36
*The American Academy of Medicine.* The Study of Medicine as a means of Education. R. L. Sibbett. 39
Medical Supervision of Student-Life. C. McIntyre ..... 40
The Climatic Treatment of Disease H. O. Marcy ..... 40
"What is Medicine?" Address by President, A. L. Gihon ..... 40
Medical Evidence. T. J. Turner ..... 42
Laws Regulating the Practice of Medicine. R. J. Dunglison, H. O. Marcy ..... 42
Health Officers. B. Lee ..... 43
Micro-Organisms. Dr. Nelson ..... 44
Relations of Bacteria to Certain Puerperal Inflammations. Dr. Cushing ..... 44
*New York State Medical Association.* Power and Right of the State to License Medical Practitioners. J. P. Gray ..... 45
State Medicine. A. L. Carroll ..... 46
Tubercular Consumption. H. Didama ..... 46
Psoitis and Pere-psoitis. S. Clark. 46
Shock. C. Brown ..... 47
Railroad Shock. F. H. Hamilton ..... 47
The Essential Nature of Shock. E. S. F. Arnold ..... 47

PAGE.

Insanity following Gun-Shot Wound. C. F. MacDonald ..... 47
Discussion on Pneumonia ..... 47
The Medico-Legal Bearing of Pelvic Injuries in Women. Dr. Van DeWarker ..... 50
Relations of Physiology to the Practice of Medicine. A. Flint, Jr. 51
Recto-Labial Fistulæ. Dr. Taylor. 51
Recurring Luxations. E. M. Moore. 51
Strangulated Oblique Inguinal Hernia. Dr. Hyde ..... 51
Chronic Intestinal Catarrh. Dr. Jamieson ..... 51
Diseases of Sullivan County. Dr. Purdy ..... 52
Enterolith. Dr. Sabin ..... 52
Commercial Prescriptions ..... 52
Prophylaxis. Dr. De Zanche ..... 52
Acne-Form Diseases. Dr. Fell ..... 52
Address on Pathology. Dr. Janeway ..... 53
Gall-Stones—Patent and Concealed. Dr. Seymour ..... 53
Theraphy of Chlorides. Dr. North. 53
Urethral Stricture. Dr. Newman ..... 54
Pelvic Hæmatocele. Dr. Seymour. 54
Thirty Cases of Diphtheria. S. W. Smith ..... 54
Peri-Uterine Hæmatoma. Dr. Harrison ..... 55
Cancer of the Kidney. Dr. Shrady. 55
Micro-Organisms and Surgical Wounds. Dr. Drunis ..... 55
*College of Physicians and Surgeons of Philadelphia.* Removal of Ovaries and Fallopian Tubes. W. W. Keen ..... 56

**Clinical Reports:**

Lupus Vulgaris. H. J. Reynolds ..... 64
Mercy Hospital. E. Andrews. Fistula of the Neck ..... 69
Chronic Synovitis of Knee ..... 71
Lupus of the Larynx. F. O. Stockton ..... 74

**State Boards of Health:**

Illinois State Board of Health ..... 77
Indiana State Board of Health ..... 79
Michigan State Board of Health ..... 80

**Book Reviews:**

Technology of Bacteria Investigation. Dolley ..... 81
Text-Book of Nursing. Weeks ..... 82
Human Osteology. Woods' Library. 83
"Restoration of Chemism." Thornton ..... 84
Pedigree of Disease. Hutchinson. 85
Acne. Bulkley ..... 86
Diseases of the Skin. Duhring ... 89

**News Items** ..... 94–96

